# Assessment of Retinal Nerve Fiber Layer Changes in Patients With Chronic Kidney Disease: A Cross-Sectional Study

**DOI:** 10.7759/cureus.105875

**Published:** 2026-03-26

**Authors:** Subhangi Sahu, Saswati Sen, Matuli Das, Siddhartha Kar

**Affiliations:** 1 Ophthalmology, Kalinga Institute of Medical Sciences, Bhubaneswar, IND

**Keywords:** chronic kidney disease, diagnosis, ganglion cell complex, optical coherence tomogram, retinal nerve fiber layer

## Abstract

Background: Chronic kidney disease (CKD) destroys kidney function over months or years, resulting in end-stage renal disease (ESRD) and contributing greatly to morbidity and mortality. The accumulation of toxins, inflammation, and reduced blood flow in CKD affects the nervous system, circulatory system, and even the eyes. The retinal nerve fiber layer (RNFL) and ganglion cell complex (GCC), being prone to the shortage of blood, are good markers for evaluating the health of the retina in diseases such as CKD. This can, in turn, aid in determining the prognosis and modifying treatment modalities in individual patients.

Purpose: To measure the RNFL and GCC thickness in patients with CKD and compare these measurements with those in age-matched healthy controls.

Methods: This comparative cross-sectional study included 100 patients with CKD and 100 age-matched healthy controls. A comprehensive ocular examination was performed for all, including best-corrected visual acuity (BCVA), intraocular pressure (IOP), and fundus assessment. Optical coherence tomography (OCT) was performed in all cases. We measured RNFL thickness (RNFLT) and GCC in all quadrants. The duration of CKD and serum urea and creatinine levels were noted. We used Pearson’s coefficient and multivariate analysis to examine the association between the parameters, with a p-value of <0.05 considered significant.

Results: The mean age was 39.39 years in the CKD group and 38.26 years in the control group. The differences in BCVA and cup-disc ratio (CDR) between the two groups were statistically significant. The mean BCVA in the control group was statistically better than that in the CKD group. The mean IOP was 11.95 ± 1.45 mmHg in the CKD group, whereas it was 15.41 ± 3.55 mmHg in the control group. The difference in RNFLT in all quadrants was statistically significant between the two groups, except in the superior quadrant. The thickness of GCC was greater in all quadrants in the control group, whereas the difference in thickness between the groups was statistically significant in all quadrants except in the inferior quadrant.

Conclusion: Substantial structural and functional alterations in the eye are present in moderate-to-severe CKD, even when elevated IOP is not present, effectively establishing a relationship between changes in the RNFL and GCC with renal function.

## Introduction

Chronic kidney disease (CKD) contributes greatly to both morbidity and mortality. It gradually destroys kidney function over a period of months or years and finally results in end-stage renal disease (ESRD) if no treatment is started. The accumulation of toxins, inflammation, stress, and reduced blood flow affects different organ systems, namely, the nervous system (both central and peripheral) and circulatory system, and even the eyes. Microvascular dysfunction and hypertension may also cause ischemia in these cases. The retinal nerve fiber layer (RNFL) consists of the axons of retinal ganglion cells, whereas the ganglion cell complex (GCC) includes the layers of ganglion cells and the inner plexiform layer that are especially susceptible to the shortage of blood and ischemic damage. Therefore, they are good markers for evaluating the health of the neural retina in systemic diseases such as CKD. Fluctuations in the thickness or texture of this layer could point to the occurrence of pathological processes at either the local or systemic level [1,2]. Research on patients with CKD has already initiated the process of revealing not very large but still important alterations in the RNFL and GCC, even before the occurrence of any major eye problem. These alterations seem to be interconnected with the time and degree of renal function loss, which may also indicate the wide-ranging microvascular and neurodegenerative processes involved in CKD [3,4]. The chronic condition of CKD usually has poor endothelial function throughout the body, leading to the formation of a weakly functional network of microcirculation that includes the blood vessels in the eye. The metabolic activity of the retina, especially the RNFL, is very high and is therefore completely reliant on having intact and well-regulated microvascular systems for both its functioning and structural preservation. Nevertheless, even with subclinical hypoperfusion and ischemia, axonal injury, and consequently RNFL damage could happen. Additionally, the presence of high blood pressure, which is seen as a cause and an effect of CKD at the same time, has been known to worsen the problem of microvascular injury and might thus cooperate with renal failure in speeding up the process of retinal changes [5,6]. The present study aims to assess the RNFL and GCC values in patients with CKD so that prognosis and disease management can be done in a better way. The estimated glomerular filtration rate (eGFR) values were also considered while assessing RNFL and GCC changes to evaluate the damage with respect to disease severity.

A part of this work was previously presented as a free paper at the Asia-Pacific Academy of Ophthalmology (APAO) Congress on February 5, 2026.

## Materials and methods

Study design

This was a comparative cross-sectional study conducted in a tertiary care setting at Kalinga Institute of Medical Sciences, Bhubaneswar, eastern Odisha, India.

Ethical considerations

We obtained Institutional Ethics Committee clearance (KIIT/KIMS/IEC/1516). The study adhered to the ethical principles of the Declaration of Helsinki. The study period was from February 2024 to November 2025.

Inclusion and exclusion criteria

The study included patients with CKD stratified by disease duration (<1 year, 1-5 years, and >5 years) and GFR stages (G3a-G5) based on eGFR levels and healthy individuals with normal renal function (eGFR >90 mL/min/1.73 m²) and no systemic/ocular diseases [7]. Matching was performed using a 1:1 ratio for age (±5 years) and sex. These subjects were mostly between 30 and 70 years and diagnosed with CKD for at least three months (in situations when the GFR was less than 60 mL/min/1.73 m² or structural kidney damage was present). We excluded patients with pre-existing glaucoma, optic neuropathy, macular edema due to diabetic or hypertensive retinopathy or any other cause, high refractive errors, pregnancy, trauma, or any other systemic or ocular disease affecting the retina. Patients with significant lens opacities hindering the visualization of the fundus were also excluded.

Sampling, study procedure, and tools

Purposive sampling was used, and we included 200 patients for analysis in the study. Consecutive patients presenting to the outpatient department (OPD) and meeting the inclusion criteria were included in the study. The CKD and control groups had 100 patients each. All these patients reported to the ophthalmology OPD of a tertiary care center in eastern Odisha. We explained the study to eligible patients and received informed consent in writing. We noted the patients' demographic profiles, took a proper clinical history, and conducted a slit-lamp examination. Parameters, such as visual acuity and intraocular pressure (IOP), measured using an applanation tonometer and fundoscopy findings were noted. Optical coherence tomography (OCT) examination was conducted to measure the RNFL thickness (RNFLT) and GCC thickness.

We used Snellen’s chart and the logarithm of the minimum angle of resolution (LogMAR) chart to record best-corrected visual acuity (BCVA), slit-lamp bio-microscopy to examine the anterior segment structures, and a +20 D condensing lens to check the dilated fundus. We used a Goldmann applanation tonometer to record the IOP and conducted OCT using a spectral domain OCT machine (Heidelberg Spectralis OCT system with Glaucoma Module Premium Edition (GMPE) and TruTrack^TM^ eye tracking; Heidelberg Engineering GmbH, Heidelberg, Germany) for measuring RNFLT and the thickness of GCC. The GCC scan was centered on the macular area using a 30-degree x 25-degree volume scan. OCT was conducted by a single experienced observer, and care was taken to avoid segmentation errors. Any scan with low signal strength was discarded, and the measurement was repeated to satisfy the required standards. The eGFR values were determined by applying the CKD Epidemiology Collaboration (CKD-EPI) equation to serum creatinine levels. Hemoglobin levels, blood pressure, and duration of CKD were included as secondary measures. Additionally, systemic biomarkers (creatinine and urea) were recorded; these were measured at least fifteen days before examination.

Statistical analysis

The IBM SPSS Statistics for Windows, Version 26 (Released 2018; IBM Corp., Armonk, New York, United States) was used for the analysis. Independent t-tests were used to compare continuous variables (RNFL and GCC), which were represented as mean ± standard deviation. We used Pearson's coefficient to examine the associations between RNFLT and eGFR/disease duration. After adjusting for age, sex, and blood pressure, the chi-square test was used for analysis. We considered a p-value less than 0.05 to be significant.

## Results

This was a cross-sectional study with 200 enrolled patients. We grouped 100 patients under the CKD group and 100 under the control group. Both eyes were examined, and only the right eye measurements were considered for the study in both groups to avoid any bias. The mean ages in the CKD and control groups were 39.39 years and 38.26 years, respectively. The number of men (46, 46%) was slightly less in the CKD group than in the control group (49, 49%), whereas there were more women in both groups. However, we found this difference to be statistically insignificant. IOP was within normal range for both groups. BCVA was better in the control group (0.13 ± 0.11) than in the CKD group (0.51 ± 0.19). The mean GCC and RNFL thickness values were greater in the control group; the value was statistically significant for RNFLT. The eGFR values were better in controls (86.36 ± 12.47) than in the CKD group (37.93 ± 7.08), and this difference was statistically significant. The details are described in Table 1.

**Table 1:** Baseline values of basic parameters ˟Independent t-test, ^#^Chi-square test used IOP: intraocular pressure; BCVA: best-corrected visual acuity; RNFL: retinal nerve fiber layer; GCC: ganglion cell complex; eGFR: estimated glomerular filtration rate; CKD: chronic kidney disease; SD: standard deviation; LogMAR: logarithm of the minimum angle of resolution

Parameters		CKD cases (N=100)	Controls (N=100)	Test statistic	p-value
Mean age (in years) (mean ± SD)		39.39 ± 12.81	38.26 ± 12.64	0.59	<0.183˟
Gender N (%)	Male	46 (46)	49 (49)	0.17	0.671^#^
	Female	54 (54)	51 (51)		
IOP (in mmHg) (mean ± SD)		11.95 ± 1.45	15.41 ± 3.55	8.34	<0.001˟
BCVA (in LogMAR) (mean ± SD)		0.52 ± 0.19	0.13 ± 0.12	16.37	<0.001˟
eGFR (in mL/min/1.73 m^2^) (mean ± SD)		37.93 ± 7.08	86.36 ± 12.47	28.99	<0.001˟
Mean RNFL (in µm) (mean ± SD)		74.25 ± 5.30	95.72 ± 6.92	22.90	<0.001˟
Mean GCC (in µm) (mean ± SD)		83.01 ± 5.00	92.36 ± 5.20	12.13	0.983˟

We mapped the four quadrants - nasal, temporal, superior, and inferior - in the OCT printout and analyzed them individually between both groups for RNFLT. The mean RNFLT was found to be lower in the CKD group (74.25 ± 5.30 µm) than in the control group (95.72 ± 6.92 µm). We found the RNFLT to be the highest in the inferior quadrant for both groups. The thickness of most quadrants was higher in the control group, and the difference was statistically significant when compared with the CKD group. The difference in thickness of all quadrants was statistically significant between the two groups, except in the superior quadrant. The details are described in Table 2.

**Table 2:** Quadrant-wise comparison of RNFL between the two groups An independent t-test was used to calculate the p-value. RNFL: retinal nerve fiber layer; CKD: chronic kidney disease; SD: standard deviation

Parameters		CKD cases (n=100) (mean ± SD)	Controls (n=100) (mean ± SD)	t	p-value
RNFL (in µm)	Nasal	105.02 ± 6.17	116.40 ± 5.70	12.83	0.012
	Temporal	66.63 ± 5.00	72.91 ± 6.18	7.47	0.017
	Superior	114.09 ± 5.98	123.99 ± 6.24	1.17	0.784
	Inferior	120.35 ± 5.58	134.70 ± 6.71	15.59	0.046

Table 3 shows a comparison of GCC values between CKD cases and the control group. Six quadrants were mapped and analyzed - superotemporal, superior, superonasal, inferotemporal, inferior, and inferonasal. Mean GCC values showed a statistically significant difference between the two groups. GCC was the highest in the superotemporal quadrant in the control group (69.88 ± 5.67) and in the inferior quadrant (63.07 ± 7.36) in patients with CKD. The thickness of all quadrants was higher in the control group as compared to CKD cases. This difference was statistically significant in all quadrants.

**Table 3:** Quadrant-wise comparison of GCC between the two groups An independent t-test was used to calculate the p-value. GCC: ganglion cell complex; CKD: chronic kidney disease; SD: standard deviation

Parameters		Controls (n=100) (mean ± SD)	CKD cases (n=100) (mean ± SD)	t	p-value
GCC (in µm)	Superotemporal	69.88 ± 5.67	57.28 ± 3.27	19.86	<0.001
	Superior	64.42 ± 6.06	58.31 ± 5.26	7.42	<0.001
	Superonasal	63.56 ± 6.18	56.40 ± 5.94	8.01	<0.001
	Inferotemporal	69.22 ± 6.14	57.30 ± 4.66	16.98	<0.001
	Inferior	65.70 ± 7.04	63.07 ± 7.36	2.61	0.010
	Inferonasal	60.44 ± 6.85	57.62 ± 4.58	4.01	<0.001

**Figure 1:**
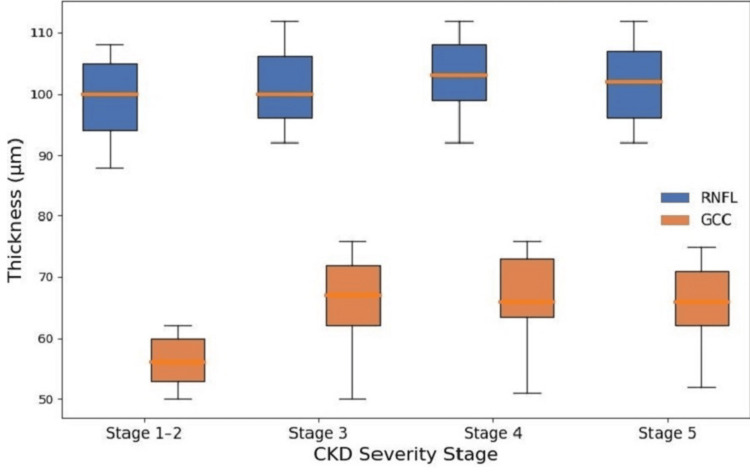
Box plot showing changes in RNFL and GCC with respect to CKD severity RNFL: retinal nerve fiber layer; GCC: ganglion cell complex; CKD: chronic kidney disease

Figure 1 depicts a box plot showing RNFLT and GCC changes in comparison to the change in the severity of CKD. When the grade of CKD was severe, the number of patients with decreased GCC values was higher compared to the number of patients with decreased RNFLT. This means that the change in GCC was more pronounced than RNFLT when compared with the disease severity. This may hold good prognostic importance while following up patients and individualizing treatment so that gross vision loss can be prevented.

## Discussion

The significance of this work lies in its potential to establish the retina as a sensitive, non-invasive biomarker of systemic microvascular and neurodegenerative injury in CKD. Inflammation has been a major theoretical axis in the conceptualization of RNFL changes in CKD. Chronic low-grade inflammation, or the presence of pro-inflammatory cytokines, such as interleukin-6, tumor necrosis factor-alpha, and C-reactive protein, characterizing CKD, is one of the main sources of the disease. It has been noted that these inflammatory factors act to loosen up the blood-retinal barriers, stimulate the glial cells, and activate the neuronal death pathways among the retinal neurons. The death of the retinal ganglion cells or their axons as a result of inflammation could lead to RNFL thinning that can be measured over the years. In addition, inflammation may further interact with oxidative stress and hence make the cell injury even worse, thereby creating a feedback loop that speeds up retinal neurodegeneration in patients with CKD [8]. Retina, being one of the highly metabolically active tissues, becomes more susceptible because advanced stages of CKD have anemia as a perturbing finding [9].

The changes in electrolytes and metabolism that come along with CKD not only have negative effects on the RNFL but also give rise to new theories about it. The constantly changing levels of calcium, phosphate, and acid-base can distort cellular signaling and homeostasis, and thus, the retinal neuron function. Common conditions of CKD, hyperphosphatemia, and secondary hyperparathyroidism may eventually cause the retinal area to become less supplied with blood due to vascular calcification. The metabolic disorders might then play a role in RNFL damage through their actions on both the blood vessels and nerves [10].

The age distribution between CKD cases and controls in the present study matched well and demonstrated significantly thinner RNFL in advanced CKD. This is in concurrence with studies conducted by Wong et al. and Sabanayagam et al., including a large cohort of patients [11,12]. Similar to the present study, Wan et al. and Singh et al. found significant RNFL loss across CKD stages without any significant gender imbalance [13,14]. This implies that age- and gender-linked variations are less likely to affect the observed differences in BCVA, IOP, RNFLT, and GCC. Similar to the difference in BCVA observed in our study, Atilgan et al. showed that 28% of patients on hemodialysis without glaucoma had localized RNFL defects and corresponding visual field changes despite normal IOP, implying that subclinical axonal loss can translate into functional impairment [15].

Global RNFLT in the present study was dramatically reduced in CKD, which far exceeds the typical age-related loss and provides strong evidence of CKD-associated neuroretinal degeneration. Comparable but somewhat smaller differences have been reported in multiple cohorts in studies conducted by Wong et al. and Fursova et al. [11,16]. The pattern of sectoral change of RNFL observed in the present study indicates preferential thinning in inferior, nasal, and temporal sectors, reminiscent of early glaucoma topography but occurring here in a normal/low IOP context. Our sectoral findings align well with the fact that temporal and inferior fibers, which subserve papillomacular and arcuate bundles, appear particularly vulnerable to CKD-related microvascular and metabolic insults, reinforcing the importance of quadrant-level OCT analysis in renal populations. Several studies have demonstrated a similar trend related to superior and inferior quadrants while comparing sectoral RNFLT [17-19].

Average macular GCC thickness was numerically lower in CKD cases than in controls, suggesting a trend toward ganglion cell loss, which was statistically significant in our dataset. However, we still cannot rule out analytic issues, sample variability, or segmentation limitations. Previous work, although less abundant for GCC than RNFL, generally supports macular involvement in CKD. Previous studies have found that CKD is associated with a smaller number of macular vessels and a larger foveal avascular zone, which suggests that the inner retinal layers, including ganglion cells, are compromised. Nevertheless, the observed GCC pattern supports the broader concept of CKD-associated inner retinal neurodegeneration and encourages combined RNFL and macular analysis in these patients [20,21].

In addition to the factors described, a comparative assessment of RNFL and GCC change in the present study has shown GCC to be slightly more sensitive to renal changes, although overall, they may not be as apt as continuous markers in advanced CKD stages.

To summarize, the possibility of RNFL alterations being indicative of the health of the whole body in a patient with CKD cannot be overlooked. RNFLT could be included as part of the routine monitoring of patients with CKD because of the relative ease of monitoring the retinal anatomy non-invasively. This might provide medical professionals with important news about the condition of the systemic microvasculature and nervous system, thus making it possible to intervene earlier. In addition, the retina gives the unique chance to monitor the disease processes in the whole body in real-time, and therefore, RNFL and GCC analyses might be used along with the traditional markers of CKD progression and complications [22].

Limitations

The cross-sectional design precludes assessment of temporal progression and causality. Hence, further longitudinal studies measuring the changes in values of RNFLT and GCC over time will give a better idea of the diagnostic value of tests. Although major ocular confounders, such as glaucoma, high myopia, and advanced retinal disease, were excluded, other systemic confounders, including detailed blood pressure profiles, nocturnal dipping status, anemia severity, dialysis adequacy indices, inflammatory markers, and lipid parameters, if considered, would strengthen the study. Multicentric studies on the subject may give a better representation of the disease burden in the community and more reliable results. In addition, segmentation errors may have led to the omission of some sectoral errors while averaging the values of RNFL and GCC, which emphasizes the need for cautious interpretation.

## Conclusions

These results suggest that substantial structural and functional alterations in the eye are present in moderate-to-severe CKD, even when elevated IOP is not present, effectively establishing a relationship between changes in the RNFL and GCC, renal function, illness duration, and fundamental ocular parameters in individuals with CKD. Future protocols should incorporate longitudinal OCT monitoring, microvascular imaging such as OCT angiography, and comprehensive systemic profiling to better understand the temporal evolution and determinants of retinal damage in CKD. It is also recommended that future studies be stratified by dialysis modality, diabetes status, and cardiovascular disease to clarify subgroup risks. Finally, integrating retinal structural markers with systemic risk scores could be explored as a way to improve prognostication for both ocular and systemic outcomes in CKD.
